# Association of single nucleotide polymorphisms of cytochrome P450 enzymes with experience of vasomotor, vaginal and musculoskeletal symptoms among breast cancer patients: a systematic review

**DOI:** 10.1186/s12885-021-08268-8

**Published:** 2021-05-18

**Authors:** Carmen W. H. Chan, Bernard M. H. Law, Marques S. N. Ng, Corinna C. Y. Wong, Carissa W. Y. Wong, Morgan Quinley, Jessica M. Orgusyan, Ka Ming Chow, Mary M. Y. Waye

**Affiliations:** 1 The Nethersole School of Nursing, Faculty of Medicine, The Chinese University of Hong Kong, Shatin, the New Territories, Hong Kong SAR, China; 2 The Croucher Laboratory for Human Genomics, The Chinese University of Hong Kong, Hong Kong SAR, China; 3 University College London School of Pharmacy, London, UK; 4 University College London Cancer Institution, University College London, London, UK; 5 Molecular, Cell & Development Biology, University of California, Santa Cruz, USA; 6 Department of Global Health, University of California, San Diego, USA; 7 Asia-Pacific Genomic and Genetic Nursing Centre, The Chinese University of Hong Kong, Hong Kong SAR, China

**Keywords:** Breast cancer patients, Adjuvant endocrine therapy, Cytochrome P450, Single nucleotide polymorphisms, Genotype, Symptom

## Abstract

**Background:**

Adjuvant endocrine therapies are known to induce undesirable adverse effects such as vasomotor, vaginal and musculoskeletal symptoms among breast cancer patients. Drugs used in these therapies are often metabolised by cytochrome P450 (CYP) enzymes, in which their metabolising activities can be modified by single nucleotide polymorphisms (SNP) in CYP genes and CYP genotypes. This review aims to explore whether SNPs or genotypes of CYP are associated with the occurrence, frequency and severity of vasomotor, vaginal and musculoskeletal symptoms in breast cancer patients on adjuvant endocrine therapies.

**Methods:**

A literature review was conducted using five electronic databases, resulting in the inclusion of 14 eligible studies, and their findings were presented narratively. Selected items from the Strengthening the Reporting of Observational Studies in Epidemiology (STROBE) checklist were used for critical appraisal of the reporting quality of the included studies.

**Results:**

Most of the included studies showed that SNPs or genotypes of CYP that modify its metabolising activity have no effect on the occurrence, frequency or severity of vasomotor symptoms, including hot flashes. One study showed no correlation of these genetic variations in CYP with musculoskeletal symptoms, and no data were available on the association between such genetic variations and vaginal symptoms.

**Conclusions:**

Overall, genetic variations in CYP have no effect on the experience of hot flashes among breast cancer patients. We recommend exploration of the link between the active metabolites of chemotherapeutic drugs and the molecules shown to affect the occurrence or severity of hot flashes, and the establishment of the relationship between such genetic variations and patients’ experience of musculoskeletal and vaginal symptoms. Subgroup analyses based on patients’ duration of adjuvant endocrine therapies in such studies are recommended.

## Introduction

Adjuvant endocrine therapies that involve the use of drugs such as tamoxifen and aromatase inhibitors (AIs), are often administered to breast cancer patients who have completed curative treatments [1]. These therapies serve to prevent the recurrence of oestrogen receptor (ER)-positive breast cancer, potentially via inhibition of oestrogen production or suppression of the tumour-inducing effects of oestrogen via its interaction with ER [2–4]. Despite evidence of the benefits of such therapies in promoting disease-free survival among breast cancer patients [5], these patients were often reported to experience undesirable symptoms that reduced their quality of life and hampered their daily functioning.

**Fig. 1:**
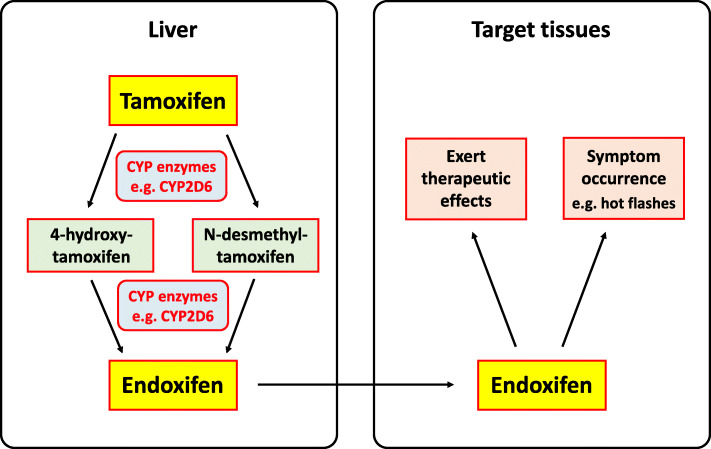
A schematic diagram showing the functions of CYP enzymes and their role in the occurrence of symptoms induced by adjuvant endocrine therapies

Vaginal atrophy, vasomotor symptoms such as hot flashes [6–8] and musculoskeletal symptoms including joint pain and stiffness [9] are commonly reported adverse effects of adjuvant endocrine therapies with tamoxifen and/or AIs. Consistent with this, a recent meta-analysis showed that the occurrence of vasomotor and musculoskeletal symptoms is associated with a lower rate of breast cancer recurrence, an outcome associated with the administration of adjuvant endocrine therapies [10]. It therefore follows that the development of the above symptoms can be attributed to the active metabolites of the drugs used in adjuvant endocrine therapies (tamoxifen and AIs). These drugs are generally metabolised in the liver by a family of enzymes called cytochrome P450 (CYP), after which the resultant metabolites then travel to the target sites to exert their therapeutic effects. For example, tamoxifen is primarily metabolised by an isoform of CYP named cytochrome P450 2D6 (CYP2D6), which eventually converts tamoxifen into the active metabolite endoxifen, although other isoforms including CYP3A4, CYP3A5, CYP2C9, CYP2C19 and CYP2B6 are also involved [11, 12]. Another CYP isoform, CYP19A1 which codes for aromatase, an enzyme involved in oestrogen synthesis, was also suggested to counteract the effects of AIs [13], which are drugs that increase the risk of vasomotor symptoms such as hot flashes. Hence, it is likely that the activity of CYP enzymes, which affects the level of metabolites produced by the drugs used for adjuvant endocrine therapy and oestrogen production, would affect the occurrence and severity of the patients’ vasomotor, vaginal and musculoskeletal symptoms. Indeed, one study showed that women with higher endoxifen levels had a greater likelihood of tamoxifen-induced symptoms such as hot flashes and vaginal dryness [14]. In support of this finding, Lee et al. [15] recently demonstrated that a dose reduction in tamoxifen treatment could reduce endoxifen levels and in turn result in patients’ perceived alleviation of hot flashes. Moreover, as indicated above, a recent meta-analysis [10] also established the relationship between the occurrence of vasomotor or musculoskeletal symptoms and breast cancer recurrence, an outcome that may be influenced by CYP’s metabolising activity [16–18], among patients who undergo endocrine therapy. Together, these findings suggest that CYP’s activities may be linked to the occurrence and/or severity of these therapy-induced symptoms. A schematic diagram depicting the functions of the CYP enzymes and their role in the occurrence of therapy-induced symptoms is shown in Fig. 1.

The known factors that affect the activities of CYP enzymes include the single nucleotide polymorphisms (SNPs) in an individual’s CYP genes and CYP genotypes [19, 20]. These SNPs and genotypes can either reduce or increase CYP’s activities, leading to modifications in metabolite or oestrogen production. For example, CYP2D6*10, an allele more commonly found among Asians [21], in which the SNP of 100 C > T occurs in CYP2D6, was found to induce a significant reduction in CYP2D6’s metabolising activity [22]. In contrast, the allele CYP2D6*2xN, caused by CYP2D6 gene duplication, can have the opposite effect [23]. In general, patients can be classified into several CYP2D6 metabolic activity classes based on the activity of the enzyme, such as poor metabolisers (PM), intermediate metabolisers (IM), extensive metabolisers (EM) and ultra-rapid metabolisers (UM). The grouping of patients into these categories depends on the CYP2D6 SNPs they possess. For example, whilst EM patients possess the normal CYP2D6 genotype, PM and IM patients possess SNPs of CYP2D6 that lead to a decrease in its metabolising activity. With previous studies suggesting a potential link between CYP activity and therapy-induced symptoms as described above, and the known effect of the SNPs of CYP on its activity [24], it is tempting to speculate that SNPs in CYP genes among these patients may affect the occurrence, frequency or severity of these symptoms, resulting in variations in symptom experience among patients. To the best of our knowledge, no recent publications have systematically reviewed studies of the relationship between the SNPs/genotypes of CYP and outcomes related to vasomotor, vaginal and musculoskeletal symptoms, which are commonly experienced by patients on adjuvant endocrine therapies. A review on this issue is warranted to help establish whether genetic variations in CYP2D6 among breast cancer patients have any effect on their symptom experience. This in turn would make a case and open avenues for further research into the development of tailored and personalised care and treatment plans for cancer patients who undergo adjuvant endocrine therapies, based on the outcomes of CYP genotyping of these patients.

Here, we conducted a systematic review to summarise the studies of the effects of SNPs in CYP on the occurrence, frequency and severity of vasomotor (hot flashes), vaginal symptoms and musculoskeletal symptoms (joint pain and stiffness) among breast cancer patients. Our goal was to determine whether an association exists between these comparators and outcomes.

## Methods

### Search strategy

A systematic search was conducted using multiple databases including PubMed, EMBASE (since 1910), APA PsycINFO (since 1806), OVID MEDLINE (since 1946) and CINAHL Complete, and studies published up to May 2020 were retrieved. The keywords involved in the search strategy reflected the type of participants, comparators and outcomes of interest indicated in the aims of the review. The combination of keywords used for the literature search is presented in Table 1. Moreover, a manual search was conducted to identify further eligible articles for inclusion by screening the reference lists of the included articles.

**Table 1:** The search strategy

“cancer” OR “carcinoma” OR “tumor” OR “malignancy”	
AND	
“CYP2D6” OR “CYP” OR “cytochrome P450”	
AND	
“polymorphism” OR “polymorphisms” OR “polymorphic” OR “genetic difference” OR “genetic differences” OR “genotype” OR “phenotype”	
AND	
“endocrine” OR “vasomotor” OR “musculoskeletal” OR “vaginal dryness” OR “vaginal atrophy” OR “vulvovaginal atrophy” OR “vaginal symptoms” OR “night sweats” OR “hot flash” OR “hot flush” OR “hot flashes” OR “hot flushes” OR “joint pain” OR “joint stiffness”	

### Eligibility criteria for inclusion

#### Criteria for participants

Studies included in the review were required to involve a sample of breast cancer patients who were either undergoing or initiating tamoxifen or AI therapies.

#### Criteria for comparators

The review included studies in which the effect of various SNPs and/or genotypes of CYP were investigated or those in which the effects of CYP metabolic activity classes such as UM, EM, IM and PM were compared.

#### Criteria for outcomes

For inclusion, studies were required to either 1) report whether the above comparators exhibited effects on the occurrence, frequency and/or severity of vasomotor symptoms (hot flashes), vaginal symptoms (including vaginal atrophy and vaginal dryness), and/or musculoskeletal symptoms (joint pain and/or stiffness), or 2) report the association between these comparators and outcomes.

#### Other criteria

Included studies were required to be original research studies that reported either prospective or retrospective cohorts, or clinical trials involving use of tamoxifen and/or AIs. Furthermore, only articles published in English were included.

### Study selection and data extraction

Four authors retrieved the citations from the aforementioned databases. After duplicates of these retrieved citations were removed, the authors screened the titles and abstracts of these citations to exclude articles that were not original research studies published in English. One author screened the abstracts of the remaining studies based on the above inclusion criteria for participants, comparators and outcomes, and the eligibility of these articles was then verified and approved by four other authors. The full texts of these eligible studies were examined to further determine their eligibility for inclusion. Disagreements between authors on eligibility were settled via discussion before data extraction.

Data extraction was conducted by four authors. The extracted data included study settings (sites of subject recruitment), sample size, patient characteristics, CYP genes examined, comparators (the independent variables), the outcomes assessed and the methods for their assessment, and the major findings of the studies. The extracted major findings were limited to those pertaining to the relationship between the comparators and outcomes defined above. After data extraction was completed, the extracted data were checked by an author independently to ensure accuracy. Any disagreements in the extracted data were discussed between the authors to achieve unanimity.

As there were variations in the methods of outcome presentation between the included studies, and in the comparators and outcomes examined, we were unable to conduct a meta-analysis on the extracted data. We therefore presented these data narratively in the form of *p* values, odds ratios, or hazard ratios with their associated 95% confidence interval, depending on the methods of outcome presentation used in each included study.

### Assessment of reporting quality of included studies

The Strengthening the Reporting of Observational Studies in Epidemiology (STROBE) checklist was used to evaluate the completeness of reporting of the methods and findings among the included studies. Ten selected items in the checklist were used for the assessment. To achieve optimal reporting quality, the studies’ authors were required to include descriptions of the study objectives, study design, sites of subject recruitment (study settings), inclusion and/or exclusion criteria of study participation, outcomes measured and methods for measurement, statistical tests used, and the participants’ characteristics. Moreover, the main results were required to be reported using estimates such as *p* values, odds ratios, or hazard ratios with their associated 95% confidence intervals, and interpretations of these results or conclusions were required. Studies were evaluated on whether each selected item was achieved, and the study’s overall reporting quality score was represented by the number of achieved items. The included studies were first distributed among four authors, who assessed their reporting quality. The ratings of each included study were then verified by another author. Disagreements in the ratings were also resolved via discussion among the authors.

## Results

### Search results

**Fig. 2:**
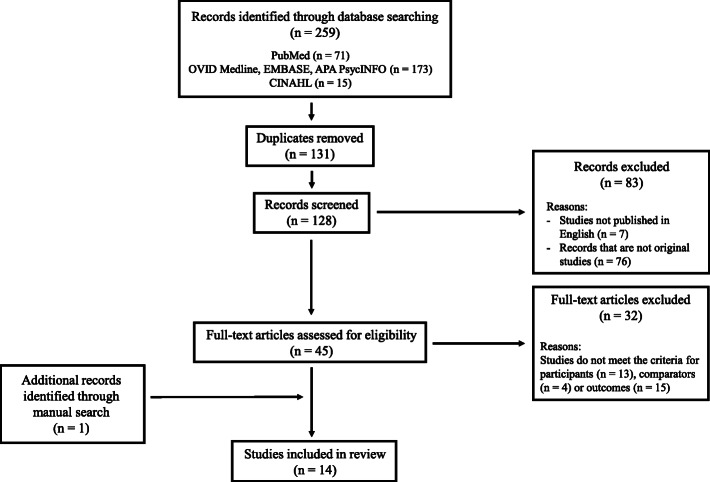
The PRISMA diagram

A total of 259 citations were identified via the literature search in the five aforementioned databases using the defined search strategy (Table 1). After the removal of 131 duplicates, the remaining 128 citations were screened for eligibility. Seven articles not published in English and 76 that did not report an original research study were removed, and the full texts of the remaining 45 articles were read and further assessed for eligibility. A further 32 articles were excluded because they failed to meet the criteria for participants (*n* = 13), comparators (*n* = 4) or outcomes (*n* = 15). Nevertheless, a manual search conducted by screening the reference lists of the included studies identified one more study that was deemed eligible for inclusion. Thus, 14 studies were included in this review. The Preferred Reporting Items for Systematic Reviews and Meta-Analyses (PRISMA) diagram depicting the search results is shown in Fig. 2.

### Critical appraisal of reporting quality of included studies

The reporting quality of the included studies ranged between moderate and excellent. With 10 being the highest score attainable, the scores assigned to these studies ranged between 5 and 10. Three of the studies fulfilled all items involved in the assessment. All studies provided the statistical methods used for data analysis and the conclusions of the study based on the interpretation of the reported findings. Most of the articles provided a clear statement of the study objectives, with the outcomes of interest in our review clearly defined and the characteristics of the study participants reported. Items that were often not fully achieved by the eligible studies include ‘study design described’, ‘study settings described’ (where the authors did not report the sites where subjects were recruited) and ‘eligibility criteria described’. Moreover, certain studies did not report statistical estimates, such as *p* values and odds ratios, when indicating whether associations exist between the comparators and outcomes, resulting in a failure to achieve the item ‘main results reported’. The critical appraisal of the included studies is summarised in Table 2.

**Table 2:** The results of the critical appraisal of the included studies

Included study	Objectives described	Study design described	Study settings described	Eligibility criteria described	Variables defined	Sources of measures described	Statistical methods described	Characteristics of participants described	Main results reported	Interpretation of results provided	Score (Max. 10)
Argalacsova et al., 2017 [25]	No	No	Yes	Yes	No	No	Yes	Yes	No	Yes	5
Baxter et al., 2014 [26]	Yes	Yes	Yes	No	Yes	Yes	Yes	Yes	Yes	Yes	9
Dezentjé et al., 2014 [27]	Yes	No	No	Yes	Yes	Yes	Yes	Yes	Yes	Yes	8
Fox et al., 2016 [28]	Yes	No	No	Yes	Yes	Yes	Yes	Yes	No	Yes	7
Goetz et al., 2005 [29]	Yes	Yes	Yes	Yes	Yes	No	Yes	Yes	Yes	Yes	9
Henry et al., 2009 [30]	Yes	Yes	No	No	Yes	Yes	Yes	No	Yes	Yes	7
Jager et al., 2013 [31]	Yes	Yes	Yes	Yes	Yes	Yes	Yes	Yes	Yes	Yes	10
Jansen et al., 2018 [32]	Yes	Yes	Yes	Yes	Yes	Yes	Yes	Yes	Yes	Yes	10
Joffe et al., 2012 [33]	Yes	No	No	Yes	Yes	Yes	Yes	Yes	No	Yes	7
Johansson et al., 2016 [34]	Yes	Yes	No	No	Yes	Yes	Yes	Yes	Yes	Yes	8
Regan et al., 2012 [35]	Yes	Yes	No	No	Yes	Yes	Yes	Yes	Yes	Yes	8
Ruddy et al., 2013 [36]	Yes	Yes	Yes	Yes	Yes	Yes	Yes	Yes	No	Yes	9
Wickramage et al., 2017 [37]	Yes	Yes	Yes	Yes	Yes	Yes	Yes	Yes	Yes	Yes	10
Zembutsu et al., 2017 [38]	Yes	Yes	Yes	No	Yes	No	Yes	Yes	Yes	Yes	8

### Characteristics of the included studies

Table 3 presents the characteristics of the included studies and their findings of interest for our review. The publication year ranges between 2005 and 2018. The single-country studies were conducted in Australia [28], Canada [26, 32], the Czech Republic [25], the Netherlands [27, 31], Sri Lanka [37] or the United States [29, 30, 33, 36]. Three further studies involved an international collaboration of research groups [34, 35, 38]. Most of the studies were cohort observational studies, although four were clinical trials [27, 28, 34, 35]. Most involved the study of the CYP2D6 isoform, although other CYP isoforms such as CYP2C9, CYP2C19, CYP2B6, CYP3A4, CYP3A5 and CYP19A1 were also investigated in some studies [26, 27, 29, 32, 34]. Ten of the 14 studies (71%) involved the comparison of the various CYP2D6 metabolic activity classes with respect to the experience of the symptoms of interest among patients. The included studies each involved hot flashes among their study outcomes, but only one study [34] involved musculoskeletal symptoms as one of the outcomes examined. None of the studies investigated the effect of SNPs and genotypes of CYP on vaginal symptoms.

**Table 3:** Characteristics of included studies and their findings of interest

Author / year / Country	Study settings	Patient characteristics / Number of patients included in analysis	Gene of cytochrome P450 genotyped and examined	Comparator	Outcomes measured	Methods of symptom assessment	Major findings relevant to review
Argalacsova et al., 2017 [25]; Czech Republic	Oncology department of a local university	Localised and advanced breast cancer patients undergoing tamoxifen therapy (*N* = 258)	CYP2D6	• CYP2D6 metabolic activity classes	• Occurrence of gynaecological adverse events including hot flashes • Time to appearance of adverse events including hot flashes	• Not specified	***Comparison between CYP2D6 metabolic activity classes*** • No significant difference was reported in the occurrence of gynaecological adverse events nor time to appearance of such events between UMs, EMs, IMs and PMs. (*p* value not reported)
Baxter et al., 2014 [26]; Canada	Personalised medicine tamoxifen clinic at a local university hospital	Breast cancer patients receiving adjuvant tamoxifen therapy (N = 132)	CYP3A4 CYP2D6	• CYP2D6 metabolic activity classes • CYP3A4 SNPs	• Hot flashes severity	• Hot flash severity survey	***Comparison between CYP2D6 metabolic activity classes*** • Genotypes of CYP2D6 that affect its metabolic activity have no correlation with hot flash severity (*p* = 0.76), after adjusting for the factor of concurrent intake of CYP2D6 inhibitory medications and tamoxifen. • However, without such adjustment, IM had significantly lower hot flash severity compared to EM (*p* < 0.01) The CYP3A4*22 SNP • Compared to patients bearing the CYP3A4 wt allele, those having the CYP3A4*22 SNP are almost 9 times more likely to have zero hot flash severity scores (OR: 8.87, 95% CI; 1.78–44.14, *p* < 0.01) • By comparing the two groups of patients (patients with wt CYP3A4 vs patients with CYP3A4*22 SNP) who have similar endoxifen levels, patients with CYP3A4*22 SNP are 13 times more likely to have zero hot flash severity score (OR: 13.0, *p* = 0.0174)
Dezentjé et al. 2014 [27]; The Netherlands	Not specified in article	Early breast cancer patients, patients to be treated with tamoxifen (*N* = 742)	CYP2D6 CYP2C9 CYP2C19 CYP2B6 CYP3A5	• SNPs of CYP2D6, CYP2C9, CYP2C19, CYP2B6 and CYP3A5 • CYP2D6 metabolic activity classes	• Hot flashes occurrence • Time to the first occurrence of hot flashes	• Adverse events case report forms	***Association between CYP SNPs and hot flashes outcomes*** • None of the CYP SNPs investigated (CYP2D6*3/*4/*6/*14/*41; CYP2C9*2/*3; CYP2C19*2/*17; CYP2B6*6/*6; CYP3A5*3) showed association with occurrence of hot flashes nor time to the first occurrence of hot flashes (*p* ≥ 0.17) ***Comparison between CYP2D6 metabolic activity classes*** • PMs appeared to be more likely to experience no hot flashes compared to IMs and EMs, but the difference in such likeliness between groups did not reach statistical significance (*p* = 0.78)
Fox et al., 2016 [28]; Australia	Not specified in article	Hormone receptor-positive breast cancer patients undergoing tamoxifen therapy (*N* = 122)	CYP2D6	• CYP2D6 metabolic activity classes	• Hot flashes frequency • Hot flashes severity	• Hot flashes diaries	***Comparison between CYP2D6 metabolic activity classes*** • Genotypes of CYP2D6 that affect its metabolic activity have no correlation with hot flash occurrence and severity (data not provided)
Goetz et al., 2005 [29]; USA	Mayo Clinic and North Central Cancer Treatment Group sites	Oestrogen receptor- positive breast cancer patients. Patients to be treated with tamoxifen followed by fluoxymesterone (*N* = 223)	CYP2D6 CYP3A5	• CYP2D6 SNPs and genotypes • CYP3A5 SNPs and genotypes	• Hot flashes severity	• Patient-reported severity of hot flashes, and graded according to the National Cancer Institute Common Toxicity Criteria (version 1)	The CYP3A5*3 SNP (6986 G > A) • No significant difference in the proportion of patients bearing the various genotypes of this variant in developing moderate or severe hot flashes (*p* value not reported) o homozygous G/G: 21% o heterozygous G/A: 25% o homozygous A/A: 17% • CYP3A5 SNPs have no association with severity of hot flashes among patients. Genotype of the CYP2D6*4 SNP (1846 G > A) • There is a tendency for a significantly higher proportion of patients bearing the homozygous wt and heterozygous genotype of this variant in developing moderate or severe hot flashes than those bearing the homozygous *4 allele. However, the difference did not reach statistical significance (*p* = 0.06). o homozygous wt/wt: 20% o heterozygous *4/wt: 23% o homozygous *4/*4: 0%
Henry et al., 2009 [30]; USA	Not specified in article	Breast cancer patients starting tamoxifen treatment (*N* = 297)	CYP2D6	• CYP2D6 metabolic activity classes	• Hot flashes frequency • Hot flashes severity	• Hot flashes diaries	***Comparison between CYP2D6 metabolic activity classes after 4 months of tamoxifen therapy*** • Significantly higher mean weekly hot flash score (indicating hot flash severity and frequency) was reported among IMs (44.3 ± 10.2), compared to EMs (26.9 ± 8.8, *p* = 0.011) and PMs (20.6 ± 16.9, *p* = 0.038). • EMs (*p* = 0.100) and PMs (*p* = 0.089) tend to have a higher likeliness in experiencing no hot flashes compared to IMs. Difference in such likeliness did not reach statistical significance. • Proportion of PMs having severe or very severe hot flashes tends to be less than that of EMs and IMs (9.5% vs 29.8%, *p* = 0.062) • Significantly higher hot flash frequency was also reported among IMs, compared to EMs and PMs (data and *p* value not reported)
Jager et al., 2013 [31]; The Netherlands	Local cancer institute	Breast cancer patients undergoing tamoxifen therapy (*N* = 109)	CYP2D6	• CYP2D6 metabolic activity classes	• Hot flashes frequency • Hot flashes severity	• Hot flashes diaries	***Comparison between CYP2D6 metabolic activity classes*** • There were no significant difference in hot flash frequency (*p* = 0.61) nor hot flash severity (*p* = 0.99) between EMs, IMs and PMs.
Jansen et al., 2018 [32]; Canada	Personalised medicine tamoxifen clinic at a local health sciences centre	Breast cancer patients undergoing tamoxifen therapy (*N* = 410)	CYP2D6 CYP3A4	• SNPs of CYP3A4 • CYP2D6 metabolic activity classes	• Hot flashes severity	• Hot flashes surveys – Patient-reported occurrence of severe hot flashes based on 7-day recall	***Comparison between CYP2D6 metabolic activity classes*** • Compared to EMs, no significant differences were observed on the level of hot flashes severity among UMs (*p* = 0.315), IMs (*p* = 0.681) and PMs (*p* = 0.822). The CYP3A4*22 SNP • The CYP3A4*22 SNP has no effect on hot flashes severity among patients (*p* = 0.762). No association between CYP3A4*22 SNP and hot flashes severity was observed.
Joffe et al., 2012 [33]; USA	Not specified in article	Breast cancer patients starting adjuvant endocrine therapy (*N* = 18)	CYP2D6	• CYP2D6 metabolic activity classes	• Occurrence of hot flashes	• Hot flashes diaries • Skin-conductance monitor	***Comparison between CYP2D6 metabolic activity classes*** • No significant difference in the proportion of patients having hot flashes induced by adjuvant endocrine therapy between the EM group and IM group (*p* value not reported).
Johansson et al., 2016 [34]; Multi-country collaboration	Not specified in article	Oestrogen receptor and/or progesterone receptor- positive early breast cancer patients before starting adjuvant therapy (*N* = 1967)	CYP19A1	• CYP19A1 SNPs	• Occurrence / Severity of hot flashes, sweating, myalgia, joint pain and joint stiffness	• Patient-reported severity of symptoms, and graded according to Common Terminology Criteria for Adverse Events Version 3.0	**Association with hot flashes** ***The rs10046 (C > T) SNP of CYP19A1*** • A significant reduction in the odds of having early-onset severe hot flashes and sweating among patients bearing the homozygous T/T genotype for this SNP, compared to those bearing the homozygous wt and heterozygous genotype. (OR = 0.78, 95% CI 0.63–0.97; *p* = 0.03) • After adjusting for confounders, reduction of such odds was still observed, but the difference did not reach statistical significance (OR = 0.83, 95% CI 0.66–1.04; *p* = 0.10) ***The rs4646 (G > T) SNP of CYP19A1*** • No significant correlation between this SNP and odds of occurrence of severe hot flashes and sweating, after adjusting for confounders (OR = 1.08, 95% CI 0.93–1.25; *p* = 0.30) **Association with musculoskeletal symptoms** ***The rs10046 (C > T) SNP of CYP19A1*** • No significant correlation between this SNP and odds of occurrence of severe musculoskeletal symptoms, after adjusting for confounders (OR = 0.84, 95% CI 0.65–1.09; *p* = 0.18) ***The rs4646 (G > T) SNP of CYP19A1*** • No significant correlation between this SNP and odds of occurrence of severe musculoskeletal symptoms, after adjusting for confounders (OR = 1.11, 95% CI 0.93–1.31; *p* = 0.25)
Regan et al., 2012 [35]; Multi-country collaboration	Not specified in article	Oestrogen receptor and/or progesterone receptor- positive breast cancer patients. Patients to be treated with tamoxifen and/or letrozole (*N* = 4393)	CYP2D6	• CYP2D6 metabolic activity classes	• Occurrence and severity of hot flashes or night sweats	• Patient-reported occurrence of hot flashes on case report forms, and severity was graded according to the Common Toxicity Criteria (version 2.0	***Among patients receiving tamoxifen who did not undergo chemotherapy previously*** • Genotypes of CYP2D6 that affect its metabolic activity are associated with the occurrence of hot flashes/night sweats (*p* = 0.02) • PMs and IMs are more likely to have hot flashes/night sweats than EMs. • Hazard ratios of developing hot flashes/night sweats: o PM vs EM: 1.24 (95% CI: 0.96–1.59) o IM vs EM: 1.23 (95% CI: 1.05–1.43) ***Among patients receiving tamoxifen who underwent chemotherapy previously*** • No association between CYP2D6 genotypes that affect its metabolic activity and hot flashes/night sweats occurrence (*p* = 0.81) • Hazard ratios of developing hot flashes/night sweats: o PM vs EM: 0.85 (95% CI: 0.47–1.54) o IM vs EM: 1.05 (95% CI: 0.76–1.45) ***Among patients receiving letrozole who did not undergo chemotherapy previously*** • No association between CYP2D6 genotypes that affect its metabolic activity and hot flashes/night sweats occurrence (*p* = 0.72) • Hazard ratios of developing hot flashes/night sweats: o PM vs EM: 1.10 (95% CI: 0.85–1.44) o IM vs EM: 0.99 (95% CI: 0.83–1.17) ***Among patients receiving letrozole who underwent chemotherapy previously*** • PMs and IMs tend to be more likely to develop hot flashes/night sweats than EMs. (*p* = 0.06) • Hazard ratios of developing hot flashes/night sweats: o PM vs EM: 1.94 (95% CI: 1.12–3.35) o IM vs EM: 1.16 (95% CI: 0.81–1.64)
Ruddy et al., 2013 [36]; USA	Oncology centre at a local cancer institute	Stage I-III breast cancer patients undergoing tamoxifen therapy (*N* = 99)	CYP2D6	• CYP2D6 metabolic activity classes (including a rare allele (RA) class – individuals in this class have an EM phenotype)	• Occurrence of hot flashes • Level of bother caused by hot flashes	• Questionnaire developed based on the Breast Cancer Prevention Trial scale of menopausal symptoms	***Comparison between CYP2D6 metabolic activity classes*** • The proportion of patients having developed hot flashes is similar between the UM/EM/RA group and IM group. (67% vs 69%) • The proportion of patients expressing that they are at least slightly bothered by hot flashes is similar between the UM/EM/RA group and IM group (63% vs 69%) • The proportion of patients expressing that they are at least moderately bothered by hot flashes is similar between the UM/EM/RA group and IM group (34% vs 38%)
Wickramage et al., 2017 [37]; Sri Lanka	Hospital clinic at the National Cancer Institute in Sri Lanka	Breast cancer patients undergoing tamoxifen treatment (*N* = 24)	CYP2D6	• SNPs of CYP2D6	• Occurrence of hot flashes	• Occurrence of hot flashes reported in clinical records	The CYP2D6*4 SNP (1846 G > A) • The existence of the CYP2D6*4 SNP in patients was not associated with the occurrence of hot flashes among them (*p* = 0.437) The CYP2D6*41 SNP (Combination of 2988 G > A, 2850 C > T and -1584C) • The existence of the CYP2D6*41 SNP in patients was not associated with the occurrence of hot flashes among them (*p* = 0.271)
Zembutsu et al., 2017 [38]; Japan and Singapore	Multi-sites; Hospitals, cancer centres and cancer institutes in Japan and Singapore	Oestrogen receptor -positive, human epidermal growth factor receptor 2-negative, invasive breast cancer patients undergoing tamoxifen therapy (*N* = 279)	CYP2D6	• SNPs/Genotypes of CYP2D6	• Occurrence of hot flashes	• Not specified	• There was no significant difference in hot flashes occurrence between patients bearing the wt CYP2D6 and those having the CYP2D6 SNPs/genotypes investigated in this study (*p* = 0.25)

*Abbreviation*: *CYP* cytochrome P450, *EM* extensive metaboliser, *IM* intermediate metaboliser, *PM* poor metaboliser, *RA* rare allele, *SNP* single nucleotide polymorphism, *UM* ultra-rapid metaboliser; wt, wild type

### Effect of SNPs of CYP on symptom experience among patients

#### Vasomotor symptoms

All 14 studies examined the relationship between SNPs and/or genotypes of CYP on the occurrence, severity and/or level of bother of vasomotor symptoms experienced by breast cancer patients (Table 3). Among them, 10 (71%) involved the comparison of the experience of such symptoms among individuals in various CYP2D6 metabolic activity classes caused by differences in the CYP2D6 gene.

Eight of the 10 studies of the various CYP2D6 metabolic activity classes reported no associations between these classes and the occurrence, frequency or severity of vasomotor symptoms, primarily hot flashes. Lending further support to this observation, Ruddy et al. [36] reported that similar proportions of individuals in the UM, EM and IM classes expressed that they were bothered by the hot flashes they experienced. In addition to the above dimensions of the outcomes, Argalacsova et al. [25] also showed that the duration before the first occurrence of the examined adverse effects, including hot flashes, did not differ significantly among individuals in the various CYP2D6 metabolic activity classes. Together, these data suggest that the SNPs of CYP2D6 that modify its metabolising activity do not affect the patients’ experience of vasomotor symptoms. Notably, Baxter et al. [26] demonstrated that the association between CYP2D6’s metabolising activity and the severity of hot flashes was absent only when the analysis excluded patients who concurrently used tamoxifen and other medications that reduce CYP2D6 activity. However, the association reappeared when this exclusion was not applied.

Two studies reported a potential effect of CYP2D6’s metabolising activity on the experience of hot flashes. Using hot-flash diaries as outcome measurements, Henry et al. [30] revealed that IMs reported greater frequency and severity of hot flashes than EMs and PMs, as evidenced by the significantly higher hot-flash scores reported by IMs after four months of tamoxifen treatment. However, the odds of experiencing no hot flashes did not differ significantly among the classes, although the proportion of PMs who experienced severe hot flashes tended to be smaller than those of IMs and EMs. Interestingly, Regan et al. [35] showed that the use of drugs for adjuvant endocrine therapy and previous curative treatment affected the experience of adverse effects including hot flashes and night sweats. In the study, patients were divided into four groups: 1) those who were undergoing tamoxifen therapy without prior chemotherapy; 2) those who were undergoing tamoxifen therapy with prior chemotherapy; 3) those who were undergoing letrozole therapy without prior chemotherapy; and 4) those who were undergoing letrozole therapy with prior chemotherapy. Among the patients who were undergoing tamoxifen therapy without prior chemotherapy, those in the IM and PM classes had significantly higher odds (> 20%; *p* = 0.02) of developing hot flashes than EMs. Nevertheless, no significant between-class differences in such odds were observed among those who received AI letrozole without prior chemotherapy (*p* = 0.72). A similar outcome was obtained when comparing the groups of tamoxifen therapy patients with and without prior chemotherapy. Likewise, although PMs and IMs tended to have higher odds of hot-flash occurrence among the group of patients undergoing letrozole therapy who had undergone chemotherapy (*p* = 0.06), such a tendency was not observed among those without previous chemotherapy (*p* = 0.72). This may be a result of the potential effect of the drugs used for adjuvant endocrine therapy and prior chemotherapeutic treatment on the development of hot flashes.

Seven studies investigated the effects of various SNPs of CYP on the experience of vasomotor symptoms among breast cancer patients. Most of these studies (71%) reported the absence of such an effect. However, Johansson et al. [34] revealed that one of the SNPs of CYP19A1, involving the rs10046 (C > T) allele, which increases enzyme activity and oestrogen production, significantly reduced the risk of severe hot flashes and sweating. Notably, individuals with the homozygous T/T genotype of CYP19A1 had a 22% reduction in this risk compared with those with the heterozygous or homozygous wild-type genotype, although this reduction no longer reached statistical significance after adjustment for confounders. Goetz et al. [29] also reported that the SNP CYP2D6*4 (1846 G > A) had the potential to affect the severity of hot flashes, as the proportion of individuals with the homozygous *4/*4 genotype for the CYP2D6 gene who developed at least a moderate level of hot flashes tended to be lower than that of individuals with the homozygous wild-type or heterozygous genotype. Nevertheless, two studies from the same research team [26, 32] reported conflicting findings as to whether the SNP CYP3A4*22 influenced the severity of hot flashes. Although the earlier study by Baxter et al. [26] reported that individuals with this SNP had significantly higher odds of reporting a zero hot-flash severity score, Jansen et al. [32] demonstrated that this SNP showed no correlation with the severity of hot flashes.

Overall, most of the included studies did not indicate a correlation or effect connecting the SNPs of CYP with the occurrence, severity and frequency of vasomotor symptoms, mainly hot flashes. Some studies showed the potential of such an association under certain circumstances, such as the use of certain drugs for adjuvant endocrine therapies and previous chemotherapy [26, 35]. However, conflicting findings hamper the drawing of firm conclusions regarding the association between the occurrence of the SNP CYP3A4*22 and development of hot flashes among patients.

#### Vaginal symptoms

None of the included studies examined the association between the SNPs of CYPs and the development, severity or frequency of vaginal symptoms, including vaginal atrophy and vaginal dryness, among breast cancer patients.

#### Musculoskeletal symptoms

The effect of the SNPs of CYP on musculoskeletal symptoms among breast cancer patients was examined in one of the included studies [34], which investigated the effect of the SNPs of CYP19A1, coding for aromatase that promotes oestrogen synthesis, on the experience of joint pain/stiffness and myalgia among patients. Two SNPs of CYP19A1 were examined (Table 3), and neither showed any significant effect on the likelihood of developing severe musculoskeletal symptoms (*p* ≥ 0.18).

## Discussion

It was previously shown that vasomotor, vaginal and musculoskeletal symptoms are among the major adverse effects associated with the use of drugs for adjuvant endocrine therapy [6–9]. Therefore, the activity of enzymes that produce the active metabolites of these drugs and those that are known to reduce the drugs’ effects, collectively known as CYPs, could affect the susceptibility of individuals to the development of these symptoms. Previous findings have shown that SNPs can influence the enzymatic activity of CYPs, so we were interested in whether these SNPs could affect, or be associated with, the occurrence of the aforementioned symptoms. Nevertheless, despite some observed heterogeneity in the study findings, our systematic review found no evidence of a relationship between the SNPs of CYPs and the occurrence, frequency and severity of vasomotor symptoms such as hot flashes. Studies that reported a significant effect of these SNPs also reported *p* values that were generally close to 0.05. These observations suggest that there is no direct effect of these SNPs on patients’ symptom experience, a finding that is consistent with some of the included studies that indicated that endoxifen levels, which are primarily regulated by the activity of CYP, have no effect on the occurrence and severity of hot flashes [31]. Nevertheless, this finding contradicts that of a recent study [15]. Moreover, we are not able to conclude whether the SNPs of CYP are associated with vaginal and musculoskeletal symptoms among breast cancer patients due to the scarcity of studies examining such associations.

Notably, the SNPs of CYP and the levels of the active metabolites of tamoxifen and AIs were not associated with the occurrence, frequency and severity of hot flashes, despite these being a common adverse effect of adjuvant endocrine therapies involving tamoxifen and AIs. Hot flashes have been suggested to be caused by an increase in the level of norepinephrine or its metabolites in the brain, as this hormone exerts its effect in the hypothalamus, inducing physiological changes pertaining to body temperature regulation such as vasodilation and sweating [39, 40]. Indeed, the intake of selective norepinephrine reuptake inhibitors, drugs that inhibit the effects of norepinephrine, was found to reduce both the frequency and severity of hot flashes [41]. With the observation herein that the SNPs of CYP have no effect on hot flashes, it is possible that tamoxifen-induced hot flashes could be induced by factors related to norepinephrine production or activity rather than directly through the effects of endoxifen. This is consistent with the indication by Regan et al. that the occurrence of hot flashes should not be used as a measure for the extent of tamoxifen metabolism [35]. As the molecular mechanisms of therapy-induced hot flashes remain largely elusive, it would be of interest to dissect the pathways by which tamoxifen can lead to development of hot flashes. Such studies would provide clues to the development of more effective therapeutic strategies reducing this therapy-induced vasomotor symptom.

Although our review revealed the lack of an effect of the SNPs of CYP on patients’ symptom experience, we highlight that the previous cancer treatment and the type of drug used for adjuvant endocrine therapy could influence the association between these two parameters. As indicated by Regan et al. [35], a significant association between CYP2D6 metabolic activity classes and the occurrence of hot flashes and/or night sweats was only observed among patients on tamoxifen therapy who had not undergone chemotherapy and not among those who had undergone chemotherapy or those on letrozole therapy. One potential explanation for the varied effect of the drugs used for adjuvant endocrine therapy on the aforementioned association could be the difference in the potential of these drugs to induce hot flashes. Previously, patients taking letrozole were reported to have fewer hot flashes than those taking tamoxifen [42, 43], and significantly fewer women undergoing letrozole therapy reported experiencing hot flashes than those on tamoxifen therapy [44]. Although largely speculative, it is possible that the comparative rarity of hot flashes experienced by patients on letrozole in Regan et al.’s study resulted in the less significant difference in the risk of developing hot flashes between various CYP metabolic activity classes in this group of patients. Likewise, prior chemotherapy was previously shown to influence the odds of tamoxifen-induced hot flashes [45]. It is possible that prior chemotherapy affected the risk of hot flashes to various extents among patients in the different CYP metabolic activity classes. The resultant large variations of the risk of hot flashes among individuals in different CYP metabolic activity classes may have contributed to the lack of significant between-class difference in the risk of this symptom’s occurrence among the subgroup of patients with prior chemotherapy. At any rate, this finding also highlights the need for subgroup analyses, based on previous cancer treatment received, in future association studies between the SNPs and genotypes of CYP and the symptom experience of patients.

Another interesting point of discussion is the possibility that SNPs of other CYP isoforms, such as CYP3A4*22, which leads to reduced drug metabolising activity of CYP3A4, are also associated with symptoms such as hot flashes among breast cancer patients. Baxter et al. [26] and Jansen et al. [32] studied whether bearing the allele CYP3A4*22 would lead to modifications in an individual’s experience of severe hot flashes. Although Baxter et al. reported significantly increased odds of experiencing no hot flashes among CYP3A4*22 carriers, the study by Jansen et al. with a larger sample size (*n* = 132 vs *n* = 410) reported a lack of an association between this SNP of CYP3A4 and the severity of hot flashes. Both studies used a hot-flash severity score for outcome assessment and were conducted by the same research group. Therefore, it is unlikely that the observed differences in study findings were due to variations in methodological approaches in data collection or ethnicity of the study sample. However, the two studies appeared to examine different outcomes. Whereas Baxter et al. primarily examined the odds of CYP3A4*22 carriers’ having a zero hot-flash severity score, which indicates the lack of this symptom, Jansen et al. investigated the effect of the CYP3A4*22 allele on the magnitude of the hot-flash severity. Therefore, although their conclusions appear contradictory, they are not necessarily mutually exclusive. It can be speculated that CYP3A4*22 carriers are indeed less likely to experience hot flashes, whilst among the patients who do experience hot flashes, those who carry the CYP3A4*22 allele do not differ in symptom severity from those who do not. Further studies on whether CYP3A4*22, or other SNPs of CYP3A4, could affect the experience of hot flashes would be worthwhile, contributing further evidence on whether CYP3A4*22 can be used as a biomarker to regulate this symptom among breast cancer patients undergoing adjuvant endocrine therapies.

Another point for discussion is the inconsistency of the findings of the included studies on whether different CYP2D6 metabolic activity classes, caused by genetic variations of the enzyme, are associated with the severity of hot flashes. Among the included studies, 10 compared the hot-flash outcomes among individuals in various CYP2D6 metabolic activity classes, of which five used the severity of hot flashes as the study outcome [26, 28, 30–32] (Table 3). Whilst Henry et al. [30] demonstrated a significant association between CYP2D6’s metabolising activity and the severity of hot flashes, the other four studies did not, except Baxter et al. [26], who showed a significant association only if concurrent intake of tamoxifen and CYP2D6 inhibitors among patients was not considered in the analysis. It is possible that this discrepancy can be explained by the difference in the time at which symptom assessment among patients was carried out. Henry et al. conducted the analysis after the patients had taken tamoxifen for four months. For the other studies, the authors reported that the patients had already been taking tamoxifen for at least eight months when they were enrolled. Therefore, the studies that reported the lack of an association involved patients who had undergone a longer duration of tamoxifen treatment, meaning that these patients were likely to have experienced therapy-induced hot flashes for a longer duration. These patients may have become more used to the experience of the symptom and therefore may not have perceived it to be so severe. With symptom assessment being a subjective measure based on patients’ perceptions, the lower perceived severity of hot flashes may have reduced the significance of the difference between the severity levels perceived by patients of various classes of CYP2D6 metabolic activity, resulting in the lack of a significant association between CYP2D6 activity and hot flashes observed in these studies. Although this speculation is yet to be proven, it suggests a need to carry out subgroup analyses of patients based on duration of tamoxifen treatment in future studies examining the association between CYP2D6 activity and symptom experience of patients.

We acknowledge several limitations of this review. First, only English-language articles were included, and it is likely that further articles reporting the outcomes of interest were thereby excluded, contributing to selection bias. Second, only studies with samples of breast cancer patients who were undergoing or will undergo adjuvant endocrine therapies were included. The reported outcomes among women at risk of breast cancer or those not on adjuvant endocrine therapies were excluded. Third, most of the included studies investigated the association of CYP2D6 metabolic activity classes with the symptom of hot flashes, and there are very limited data on the effect of SNPs and genotypes of various CYP isoforms on other symptoms induced by adjuvant endocrine therapies such as musculoskeletal and vaginal symptoms. Fourth, the level of the symptoms examined by the included studies are largely of a subjective nature. It is therefore difficult to make any fair comparisons on the effect of the examined SNPs and genotypes of the CYP gene on the severity of these symptoms between these studies, and a meta-analysis on such effect is impossible. Such difficulty also imposes challenges to the evaluation on whether these SNPs would elicit various levels of symptom burden among the patients. Finally, a number of the included studies utilised small sample sizes, which may have led to the apparent lack of significant effect of the examined SNPs and genotypes on the assessed symptoms in this review.

### Implications for further research

Given the observable link between adjuvant endocrine therapies among breast cancer patients and their experience of vasomotor, vaginal and musculoskeletal symptoms, further research on the molecular mechanisms by which these symptoms are linked to such therapies is warranted. This would provide a clearer picture of how these therapies contribute to the occurrence of these symptoms, which in turn would provide clues to the discovery of molecular pathways that can be targeted in development of therapeutic options for symptom management. Most of the studies included in the review showed a lack of evidence for a correlation between SNPs or metabolic activity classes of CYP and the vasomotor symptoms induced by adjuvant endocrine therapies among breast cancer patients. Therefore, it appears unlikely that such symptoms can be directly attributed to CYP activity. Research efforts may be directed towards the exploration of the link between the active metabolites of the therapeutic drugs and the molecules shown to affect the occurrence or severity of hot flashes, such as norepinephrine [46] and oestrogen [40]. Studies should investigate whether changes in the level of these metabolites could affect the activities of these hormones, thereby yielding clues as to whether adjuvant endocrine therapies could lead to the development of the aforementioned symptoms through signalling via these molecules.

Moreover, few studies have assessed adjuvant endocrine therapy-induced vaginal and musculoskeletal symptoms. Given their potential negative impact on patients’ quality of life [47], more research may also be conducted on whether genetic variations of CYP genes are associated with these symptoms. Such research could provide an evidence base to judge whether targeting the effect of certain genetic variations of CYP could be effective in alleviation of vaginal and musculoskeletal symptoms, thereby providing useful clues for the development of strategies for the management of these symptoms. Likewise, further research of other genetic polymorphisms such as oestrogen receptor [27] and tachykinin receptor 3 loci [48] should also be considered, which might reveal association of hot flashes with tamoxifen administration in breast cancer patients.

Finally, given the potential subjectivity of symptom assessment and the possible decline in perceived symptom severity by patients during their treatment, future studies of the associations between genetic variants of CYP and symptom severity may consider performing subgroup analyses based on groups of patients with different duration of treatment. For clinical trials in which such subgroup analyses are performed, longitudinal assessment of the possible association may be conducted by assessing symptoms at various time points of the treatment regimen. This would enable a more reliable assessment of the potential association and help establish whether the duration of treatment affects the significance of this association.

## Conclusions

Overall, our review did not find evidence for an association between SNPs/genotypes of CYP and the occurrence, frequency or severity of vasomotor symptoms such as hot flashes among breast cancer patients who are undergoing adjuvant endocrine therapies. Most studies reported the lack of an association between these comparators and vasomotor symptoms. Moreover, the small number of studies of the link between a particular SNP and a particular outcome of symptom experience, such as the effect of CYP3A4*22 on hot flashes, makes it difficult to determine whether such SNPs are associated with patients’ experience of hot flashes. Likewise, given the paucity of available data linking various SNPs/genotypes of CYP with musculoskeletal and vaginal symptoms, we could not establish the effect of these SNPs/genotypes of CYP on these two symptom types. However, it is important to note that the lack of an observed effect of SNPs/genotypes of CYP does not necessarily imply that the difference does not exist, given that some studies did report a significant effect of these SNPs or genotypes on the severity and/or frequency of patients’ vasomotor symptoms. Further research on such effect is recommended. Here, we recommend that further studies should explore the link between the active metabolites of the therapeutic drugs and the molecules shown to affect the occurrence or severity of hot flashes, such as norepinephrine and oestrogen. Further studies may be conducted to address the question of whether genetic variations of CYP could modify patients’ experience of musculoskeletal and vaginal symptoms. For these studies, subgroup analyses may be valuable. Moreover, more studies should investigate the potential of CYP3A4*22 in modifying the experience of hot flashes among breast cancer patients who are undergoing adjuvant endocrine therapies. These studies could provide further insights into the development of strategies for the personalisation of care plans for effective symptom management among breast cancer patients who are undergoing adjuvant endocrine therapies.

## Data Availability

Not applicable.

## Abbreviations

AI: Aromatase inhibitor
CYP: Cytochrome P450
CYP2D6: Cytochrome P450 2D6
EM: Extensive metaboliser
ER: Oestrogen receptor
IM: Intermediate metaboliser
PM: Poor metaboliser
PRISMA: Preferred Reporting Items for Systematic Reviews and Meta-Analyses
SNP: Single nucleotide polymorphisms
STROBE: Strengthening the Reporting of Observational Studies in Epidemiology
UM: Ultra-rapid metaboliser
